# Both lipopolysaccharide and lipoteichoic acids potently induce anomalous fibrin amyloid formation: assessment with novel Amytracker™ stains^†^

**DOI:** 10.1098/rsif.2017.0941

**Published:** 2018-02-14

**Authors:** Etheresia Pretorius, Martin J. Page, Lisa Hendricks, Nondumiso B. Nkosi, Sven R. Benson, Douglas B. Kell

**Affiliations:** 1 Department of Physiological Sciences, Stellenbosch University, Stellenbosch Private Bag X1, Matieland 7602, South Africa; 2 School of Chemistry, The University of Manchester, 131 Princess Street, Manchester, Lancs M1 7DN, UK; 3 The Manchester Institute of Biotechnology, The University of Manchester, 131 Princess Street, Manchester, Lancs M1 7DN, UK; 4 Centre for Synthetic Biology of Fine and Speciality Chemicals, The University of Manchester, 131 Princess Street, Manchester, Lancs M1 7DN, UK

**Keywords:** lipopolysaccharide, lipoteichoic acids, iron, conjugated oligothiophene dyes, thioflavin T, amyloidogenesis, fibrin

## Abstract

In recent work, we discovered that the presence of highly substoichiometric amounts (10^−8^ molar ratio) of lipopolysaccharide (LPS) from Gram-negative bacteria caused fibrinogen clotting to lead to the formation of an amyloid form of fibrin. We here show that the broadly equivalent lipoteichoic acids (LTAs) from two species of Gram-positive bacteria have similarly (if not more) potent effects. Using thioflavin T fluorescence to detect amyloid as before, the addition of low concentrations of free ferric ion is found to have similar effects. Luminescent conjugated oligothiophene dyes (LCOs), marketed under the trade name Amytracker™, also stain classical amyloid structures. We here show that they too give very large fluorescence enhancements when clotting is initiated in the presence of the four amyloidogens (LPS, ferric ions and two LTA types). The staining patterns differ significantly as a function of both the amyloidogens and the dyes used to assess them, indicating clearly that the nature of the clots formed is different. This is also the case when clotting is measured viscometrically using thromboelastography. Overall, the data provide further evidence for an important role of bacterial cell wall products in the various coagulopathies that are observable in chronic, inflammatory diseases. The assays may have potential in both diagnostics and therapeutics.

## 1. Introduction

In recent work, we have developed the idea that lipopolysaccharides (LPS) from the Gram-negative cell envelope can be shed from dormant bacteria or from continual bacteria entry into the blood, e.g. via a leaky gut, and serve to contribute to the chronic inflammation characteristic of a considerable number of diseases [1–6]. Coupled to iron dysregulation [7], this leads to various coagulopathies [8] and changes in the morphology [9] both of erythrocytes and of the fibrin formed following blood clotting. A particularly striking finding was the fact that this ‘anomalous’ fibrin (sometimes referred to by us as ‘dense matter deposits' [10,11]) could be induced by tiny amounts of LPS amounting to one LPS molecule per 10^8^ molecules of fibrinogen [12]. This kind of substoichiometric or autocatalytic activity was reminiscent of prion-like or β-amyloid behaviour. Indeed, the ‘anomalous’ fibrin was found to be stainable by the amyloid-selective stain thioflavin T (ThT), and hence amyloid in nature [12]. This provided a straightforward explanation for a number of elements of its biology, not least its resistance to degradation by the usual enzymes [8,11].

This narrative could account for the role of Gram-negative bacteria, but not for that of Gram-positives, as these do not possess LPS. By contrast, their cell walls contain lipoteichoic acids (LTAs), soluble peptidoglycan and muropeptides. There is a general feeling [13], especially because LTA has been properly purified [14], that LTA is just as capable as is LPS of providing an inflammatory stimulus to cells. While LPS mainly interacts with toll-like receptor 4 [2,15–17], LTA stimulates target cells mainly by activating toll-like receptor 2 [13,18–28], and with the glycolipid anchor of LTA playing a central role, analogous to the lipid A of LPS [29]. This is reasonable, as from the host's point of a view an invading microorganism is simply undesirable, whatever its reaction to the Gram stain. Indeed, modulo a few detailed differences [30], and certainly for our present purposes, it seems that LTA is indeed broadly equivalent to LPS in terms of its ability to stimulate an innate immune response [31–33].

**Figure 1.**
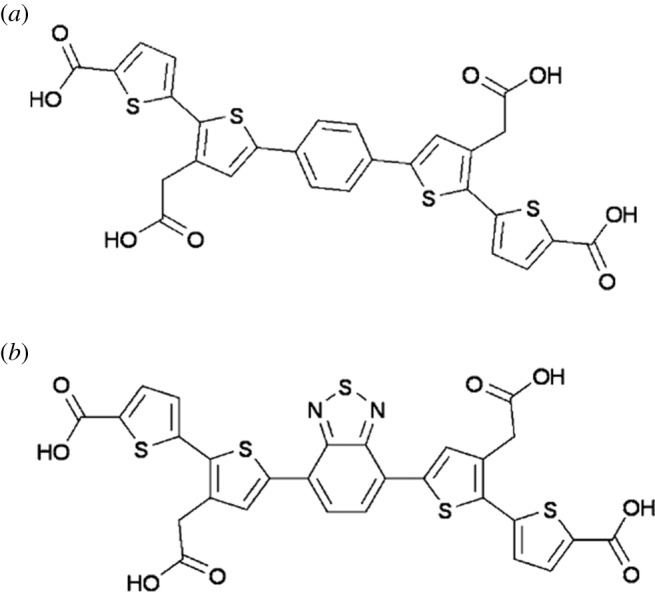
Chemical structures and in one case SMILES [77] representation of (a) HS163-SMILES (OC(_•_O)CC1 _•_ C(SC(_•_C1)C1 _•_ CC _•_ C(C _•_ C1)C1 _•_ CC(CC(O)_•_O)_•_ C(S1)C1 _•_ CC _•_ C(S1)C(O)_•_O)C1 _•_ CC _•_ C(S1)C(O)_•_O) and (b) HS169 (structures taken from [78]).

As well as the well-known ThT (e.g. [11,34–50]), a considerable number of other fluorogenic stains have been shown to illuminate amyloids (e.g. [11,51–67]). In particular, Nilsson *et al*. have developed a number of novel fluorescent amyloidogenic markers. Some of these are referred to as luminescent conjugated oligothiophenes (LCOs) [68–76] and are marketed as Amytracker™ 480 and 680 (derived, respectively, from HS163 and HS169 in the literature [71,74]), but proprietary structures that are not identical to them; figure 1 for the chemical structures of HS163 and HS169). Although they too show strong selectivity for, and enhanced fluorescence when binding to, classical amyloid proteins, their staining properties clearly differ from those of ThT [68,70,79–81]. In some cases, their binding affects prion formation itself [82,83] (and even ThT has anti-ageing properties at low concentrations [84]). It was thus of interest to assess these too as amyloid markers of the fibrin(ogen) blood clotting system.

The question then arose as to whether LTA and unliganded iron (which is also almost always dysregulated during inflammation [7,85,86]) could display just as strong an ability to divert fibrinogen polymerization from the normal to the amyloid form as could LPS. This was very much the case, and the present study shows the amyloid-forming nature of blood clots formed in plasma in the presence of low concentrations of iron, LPS from *Escherichia coli* and two LTAs from *Staphylococcus aureus* and *Streptococcus pyogenes.* The latter were chosen on the basis of coming from infectious Gram-positive organisms.

## 2. Material and methods

### 2.1. Sample population

Forty healthy individuals were included in the study. Exclusion criteria for the healthy population were known (chronic and acute) inflammatory conditions such as asthma, human immunodeficiency virus or tuberculosis; risk factors associated with metabolic syndrome; smoking; and, if female, being on contraceptive or hormone replacement treatment. This population did not take any anti-inflammatory medication. Whole blood (WB) of the participants was obtained in citrate tubes. WB was used for thromboelastography (TEG) [8,87] and platelet poor plasma (PPP) was used for confocal and super-resolution analysis.

### 2.2. Iron, lipopolysaccharide, LTA1 and LTA2

The following concentrations of the various amyloid-inducing substances were used.

— A final exposure iron concentration (FeCl_3_, MW 270.32) of 5 µM was used in all experiments.
— The LPS used was from *E. coli* O111:B4 (Sigma, L2630). A final LPS exposure concentration of 0.4 ng l^−1^ was used.
— LTA1 was from *S. aureus* (Sigma, L2515) and a final LTA1 exposure concentration of 1 ng l^−1^ was used in all experiments.
— LTA2 was from *S. pyogenes* (Sigma, L3140) and a final LTA1 exposure concentration of 1 ng l^−1^ was used in all experiments.
— LTA1P: owing to the possibility that commercial LTA (see source above) could possibly be LPS-contaminated, we also obtained a purified version of LTA1 (LTA-SA from *S. aureus*) and endotoxin-free water from Invivogen (#15E011-MM). We exposed two of our PPP naive samples to this LTA at 1 ng l^−1^. We also reduced the concentration to 0.5, 0, 25 and 0.125 ng l^−1^ to determine if we can dilute the amyloid effect to extinction.

### 2.3. Confocal microscopy

PPP was prepared by centrifuging WB for 15 min at 3000*g*, followed by storage at −80°C. On the day of analysis, all −80°C-stored PPPs were brought to room temperature and incubated for 1 h with the four candidate amyloidogenic molecules (either LPS, iron, LTA1 or LTA2 (final concentrations given in the previous section)) before adding fluorescent markers. This pre-incubation was followed by an incubation of 30 min with ThT at a final concentration of 5 µM and Amytracker™ 480 and 680 (0.1 µl into 100 µl PPP). Naive PPP was incubated only with the three markers. Before viewing clots on the confocal microscope, thrombin was added in the ratio 1 : 2 (5 µl thrombin: 10 µl PPP) to create extensive fibrin networks. Thrombin was provided by the South African National Blood Service, and the thrombin solution was at a concentration of 20 U ml^−1^ and made up in a biological buffer containing 0.2% human serum albumin. A coverslip was placed over the prepared clot, and samples were viewed using a Zeiss LSM 780 with ELYRA PS1 confocal microscope with a Plan-Apochromat 63x/1.4 Oil DIC objective. For ThT, the excitation laser used was 488 nm and emission measured at 508–570 nm; for Amytracker™ 480, the 405 nm excitation laser was used with emission measured at 478–539 nm; and for Amytracker™ 680, the 561 nm excitation laser was used for excitation with emission measured at 597–695 nm. A selection of micrographs of the prepared clots with and without the four molecules was captured. Gain settings were kept the same during all data capture and used for statistical analyses; however, brightness and contrast were slightly adjusted for figure preparation. We also prepared *Z*-stacks of clots where the candidate amyloidogenic molecules (iron, LPS, LTA1 and LTA2) were added to PPP.

We also exposed LTA1P (Invivogen purified LTA from *S. aureus*) to two healthy naive PPP samples to determine amyloid formation. Our exposure concentration ranges were 1 ng l^−1^, and we reduced the concentration to 0.5, 0.25 and 0.125 ng l^−1^ to determine if we can dilute the amyloid effect to extinction.

### 2.4. Thromboelastography

#### 2.4.1. Clot property studies

WB was incubated for 24 h at room temperature with either iron, LPS, LTA1 or LTA2, or left untreated (naive sample). Clot property studies using TEG were performed as follows: 340 µl of naive or treated WB was placed in a TEG cup and 20 µl of 0.2 M CaCl_2_ was added. CaCl_2_ is necessary to reverse the effect of the collecting tube's sodium citrate and consequently initiate coagulation. The samples were then placed in a Thromboelastograph 5000 Hemostasis Analyzer System for analysis. Seven parameters, as shown in table 1, were studied [87–89].

**Table 1.** TEG clot parameters for WB and PPP (taken from [87]).

parameters	explanation
*R* value: reaction time measured in minutes	time of latency from start of test to initial fibrin formation (amplitude of 2 mm); i.e. initiation time
*K*: kinetics measured in minutes	time taken to achieve a certain level of clot strength (amplitude of 20 mm); i.e. amplification
*Α* (alpha): angle (slope between the traces represented by *R* and *K*); measured in degrees	the angle measures the speed at which fibrin build up and cross-linking takes place, hence assesses the rate of clot formation; i.e. thrombin burst
MA: maximal amplitude measured in mm	maximum strength/stiffness of clot. Reflects the ultimate strength of the fibrin clot, i.e. overall stability of the clot
maximum rate of thrombus generation (MRTG) measured in dyn cm^−2^ s^−1^	the maximum velocity of clot growth observed or maximum rate of thrombus generation using *G*, where *G* is the elastic modulus strength of the thrombus in dyn per cm^−2^
time to maximum rate of thrombus generation (TMRTG) measured in minutes	the time interval observed before the maximum speed of the clot growth
total thrombus generation (TTG) measured in dyn cm^−2^	the clot strength: the amount of total resistance (to movement of the cup and pin) generated during clot formation. This is the total area under the velocity curve during clot growth, representing the amount of clot strength generated during clot growth

TEG results were analysed using both the paired *t*-test and the one-way analysis of variance (ANOVA) Dunnett's multiple comparisons tests, where we compared the mean of each column to the mean of the control column. The paired *t*-test was performed with STATSDIRECT (v. 2.8.0) software, and the ANOVA was performed with GraphPad Prism 7. We chose to do both analyses, as the paired *t*-test compares the naive sample with its corresponding and matched exposure sample. We additionally performed the one-way ANOVA on the full dataset; however, one of the assumptions of the ANOVA is that the groups being compared are independent, and our sample sizes between our groups are different [90]. The ANOVA is thus not the most appropriate test for this study design, as the experiments are on dependent groups. One of the primary reasons for employing the one-way ANOVA is to control for statistical error that may arise from performing multiple *t*-tests on the same sample group. Notwithstanding its arbitrary nature [91–93], significance was taken as *p* ≤ 0.05.

Confocal techniques are usually used only as qualitative methods. We captured the fluorescent signal of each of the three fluorescent markers as a composite.czi file in the Zeiss ZEN software and then used ImageJ (FIJI) to split the channels. Then we assessed the variance between (black) background and the presence of fluorescent pixels (binary comparison) for each of the three fluorescent markers in clots. For this, we used the histogram function in ImageJ (FIJI) and calculated the coefficient of variation (CV) (as s.d./mean) as our metric to quantify and discriminate between clots of healthy naive PPP and clots with the candidate amyloidogenic molecules. Sample analysis was performed with the one-way ANOVA and Dunnett's multiple comparisons test, using the GraphPad Prism 7 software.

## 3. Results

### 3.1. Confocal microscopy

**Figure 2.**
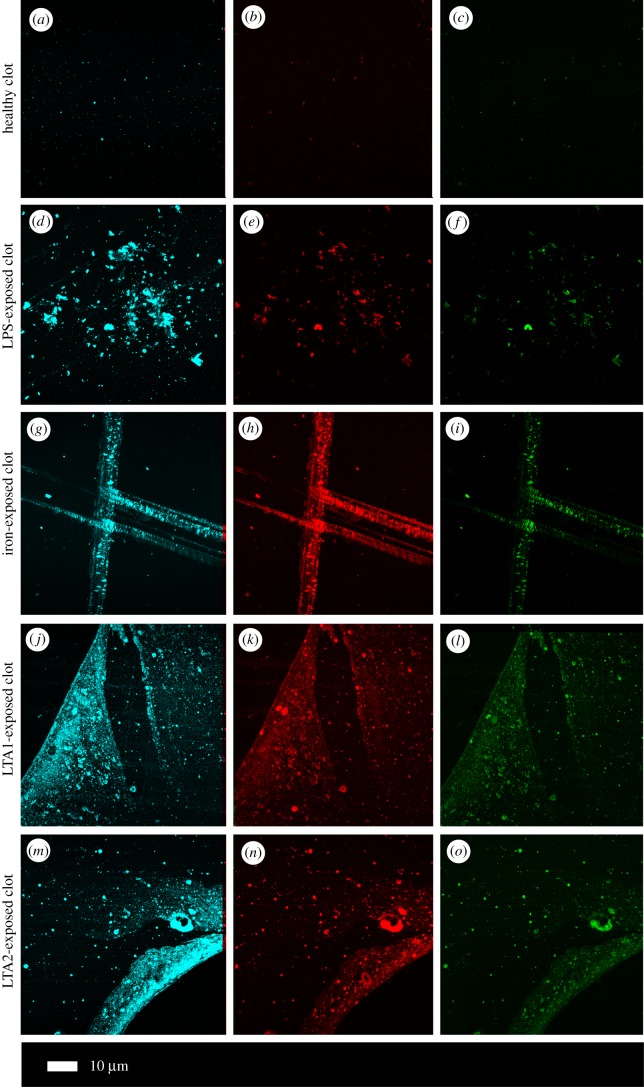
(*a*) Representative confocal images of three markers (cyan: Amytracker™ 480; red: Amytracker™ 680; green: ThT). The following micrographs are representative of the various exposures: (*a*–*c*) naive PPP; (*d*–*f*) PPP exposed to LPS; (*g*– *i*) PPP exposed to iron; (*j*–*l*) PPP exposed to LTA1; (*m*–*o*) PPP exposed to LTA2.

**Figure 3.**
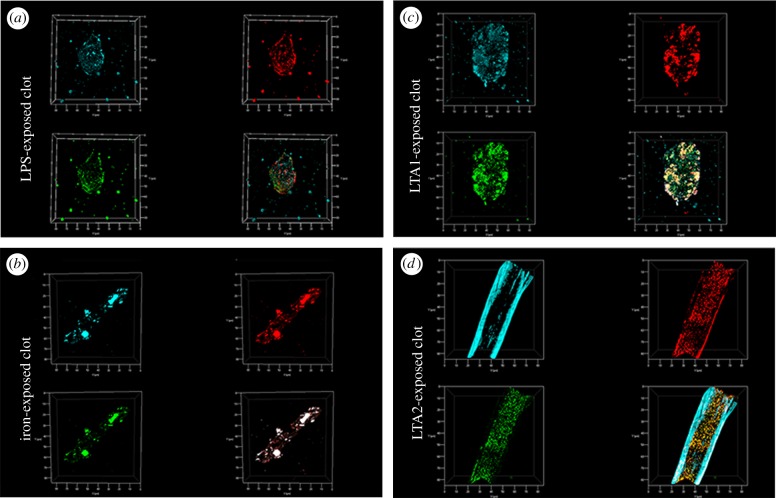
*Z*-stack projections were created with confocal microscopy and ZEN software by adding four candidate amyloidogenic molecules to naive plasma. From top left clockwise, each figure shows Amytracker™ 480 (cyan); Amytracker™ 680 (red) and ThT (green). Bottom right shows the composite of the three markers. Note that in some instances, the composite shows white areas; these areas are where all three markers overlap. (*a*) Clots of PPP exposed to LPS; (*b*) clots of PPP exposed to iron; (*c*) clots of PPP exposed to LTA1; (*d*) clots of PPP exposed to LTA2.

**Figure 4.**
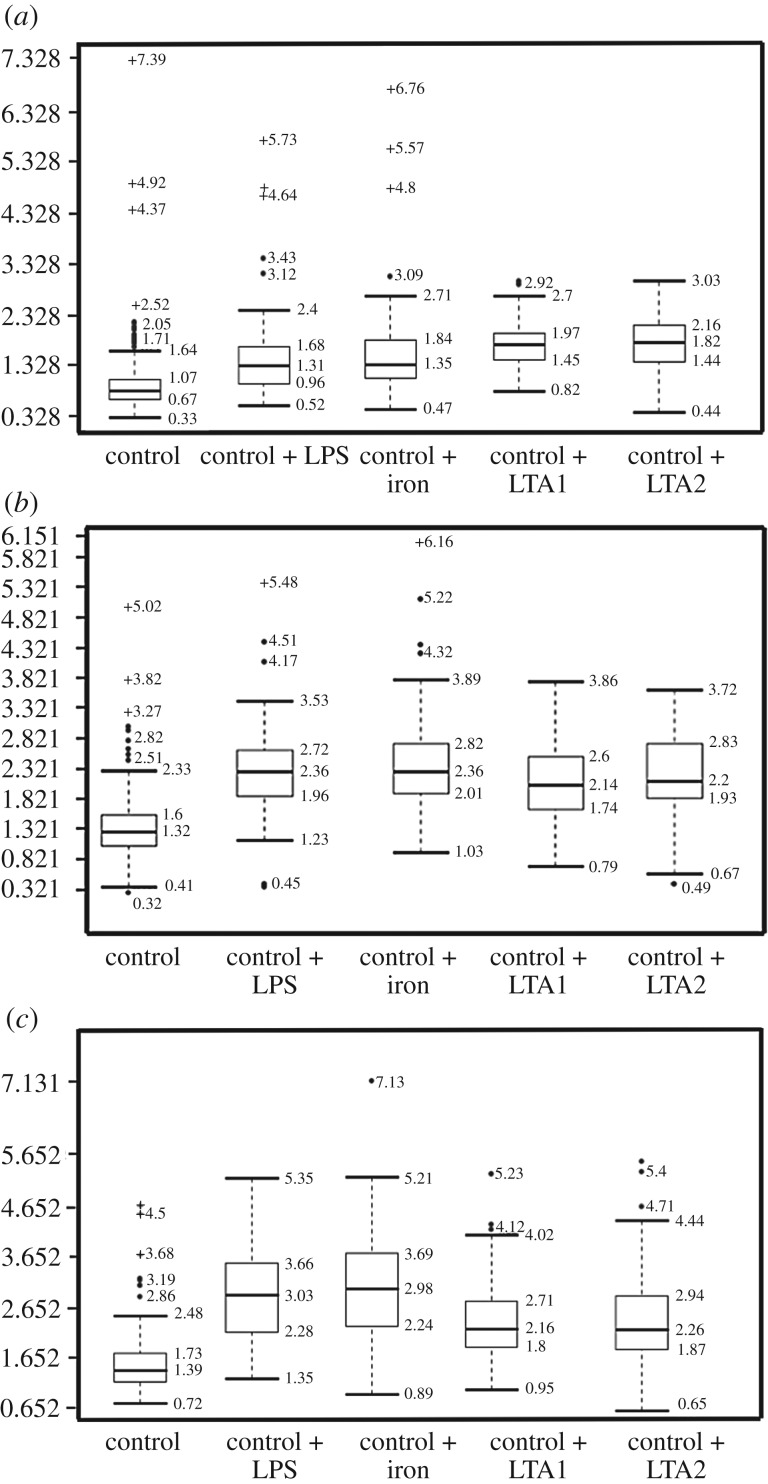
Boxplot of the distribution of the CV for the pixel intensities in the confocal clot images from the three different markers analysed (median coefficients of variation and STDs for each group are reported above the plots). (*a*) Amytracker™ 480, (*b*) Amytracker™ 680, (*c*) ThT. Data from the three different markers of each of the four molecules all differed significantly from that of the controls (*p* < 0.0001).

Confocal analysis of healthy clotted naive PPP, in the presence of ThT, Amytracker™ 480 and 680, showed occasional small patches of fluorescence (figure 2*a–c*). However, when any of the four candidate amyloidogenic molecules were pre-incubated with healthy PPP prior to addition of thrombin, the fluorescence in all three channels was greatly enhanced. This suggests increased binding of ThT, as well as of the two Amytracker markers. Amytracker binding, in particular, is a confirmation that amyloidogenesis is promoted by exposure to the four molecules. Amyloidogenesis was most prominent in PPP exposed to the two LTAs, suggesting that there are increased β-sheet-rich amyloid areas in the LTA-exposed fibrin(ogen) (figure 2*j*–*o*). Previously, we concluded that LPS binding causes the fibrinogen to polymerize into a form with a greatly increased amount of β-sheet (in the presence of thrombin), reflecting amyloid formation [12]. This results in a strong fluorescence observable (when excited at approx. 440 nm) in the presence of ThT (see e.g. [11,35,36,49,94]). Here, we confirm that the LPS, iron and the two LTAs not only result in ThT binding, plausibly to open hydrophobic areas on fibrin, but that these molecules can indeed initiate amyloidogenesis of fibrin(ogen) (as confirmed by the Amytracker™ 480 and 680 binding). The analysis of the micrographs suggests that the Amytracker™ 480 and 680 and the ThT do not bind at identical molecular sites on the fibrin, but that they bind in the same molecular vicinity. We therefore suggest that ThT and Amytracker™ binding do not interfere with each other. Figure 3*a*–*d* also shows separate and composite z-stack figures of healthy PPP exposed to the four different molecules. These results suggest that the Amytrackers™ mainly bind on different parts of the proteins, and that their binding pattern differs between the amyloid formed in the presence of the four different amyloidogenic molecules. Videos of the *z*-stacks are stored with raw data on OneDrive. Statistical (CV) data are plotted in figure 4. Data from the three different markers of each of the four amyloidogenic molecules all differed significantly from that of the controls (*p* < 0.0001) (one-way ANOVA with Dunnett's multiple comparisons tests). CV analyses were done on over 1750 micrographs.

**Figure 5.**
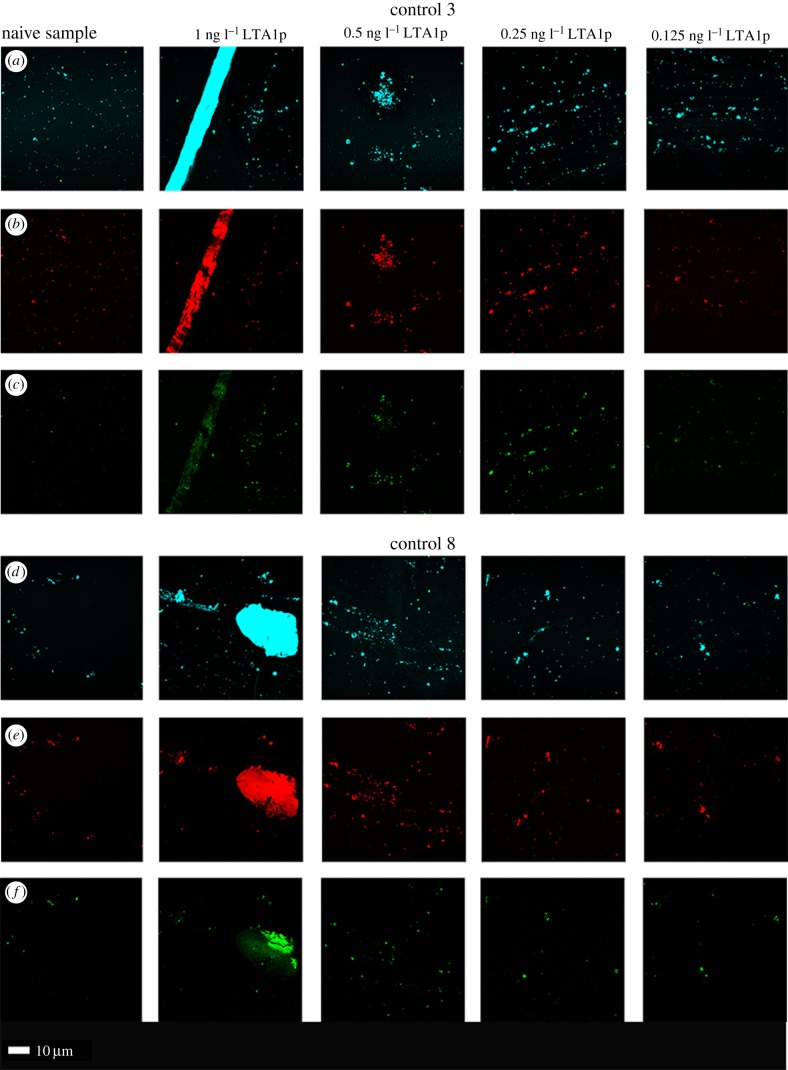
Confocal analyses where different concentrations of purified (LTA-SA from *S. aureus* (LTA1P) were added to two healthy naive PPP (left column)). Final exposure concentrations were 1, 0.5, 0.25 and 0.125 ng l^−1^ to determine if we can dilute the amyloid effect to extinction. Note that the lowest concentration appeared very like the naive sample, as naive samples do have an amyloid footprint. (*a*,*d*) Amytracker 480; (*b*,*e*) Amytracker 680; (*c*,*f*) ThT.

Confocal analyses where we added LTA1P to two healthy naive PPP samples are shown in figure 5. Our final exposure concentrations were 1, 0.5, 0.25 and 0.125 ng l^−1^ to determine if we can dilute the amyloid effect to extinction. The confocal results obtained with LTA1 and LTA1P experiments showed similar patterns of amyloid formation, and a dose–response could be seen. However, it should be noted that even in naive samples, some amyloid is present.

### 3.2. Thromboelastography

Tables 2 and 3 show demographic data of the sample (used for confocal and TEG analysis) and the TEG results of the naive WB, as well as the results after 24 h exposure with the four different molecules. We used both paired *t*-tests and one-way ANOVA analysis. In the current experiments, in some cases, there is overlap of samples, and only the appropriate subsets were used in a direct paired *t*-test analysis. Our paired *t*-test results showed that there is a significantly decreased *R*-value in the presence of LPS, suggesting that the LPS causes WB to clot faster [87]. This was also previously demonstrated with a lower LPS concentration.

**Table 2.** Demographics and TEG results of naive blood versus LPS, iron, LTA1 and LTA2. Data in the table show median ± s.d. for full dataset (*n*-value of sample size in table header) of TEG parameters for the particular exposure. *p*-values were calculated by a paired *t*-test using only the corresponding naive sample. Bold represents *p* ≤ 0.05.

healthy individuals (*N* = 40)									
gender					age				
55% F; 45% M					32.5 (±18.8)				

TEG results naive whole blood after 24 h exposure to LPS, iron, LTA1 and LTA2									
	naive (*n* = 32)	LPS (*n* = 30)	*p*-value (naive/LPS)	iron (*n* = 27)	*p*-value (naive/iron)	LTA1 (*n* = 15)	*p*-value (naive/LTA 1)	LTA2 (*n* = 15)	*p*-value (naive/LTA 2)
*R*	9.7 ± 1.8	8.5 ± 1.6	**0.02**	7.1 ± 1.1	**<0.0001**	9.2 ± 1.6	0.50	8.3 ± 1.6	0.25
*K*	3.1 ± 0.9	3.4 ± 1.0	0.34	2.7 ± 0.8	**0.003**	2.7 ± 0.8	0.07	2.7 ± 0.9	0.14
angle	50.7 ± 7.8	47.6 ± 10.0	0.18	53.7 ± 7.8	**0.0004**	55.9 ± 8.3	0.11	54.2 ± 8.7	0.97
MA	59.0 ± 7.9	57.9 ± 5.8	0.38	57.9 ± 6.0	0.61	59.4 ± 7.3	0.26	60.8 ± 6.2	**0.007**
MRTG	4.22 ± 1.43	4.13 ± 1.26	0.29	4.39 ± 1.51	0.11	5.04 ± 2.60	0.16	5.49 ± 1.82	0.11
TMRTG	13.42 ± 2.70	12.54 ± 2.72	0.17	10.17 ± 1.67	**<0.0001**	12.25 ± 2.23	0.19	11.58 ± 2.55	0.26
TTG	729.8 ± 247.68	689.69 ± 163.71	0.12	702.28 ± 170.15	0.36	729.95 ± 246.81	0.40	779.91 ± 205.04	**0.014**

**Table 3.** TEG results of naive blood versus LPS, iron, LTA1 and LTA2, analysis using ANOVA Dunnett's multiple comparisons tests. Bold represents *p* ≤ 0.05.

TEG results of naive whole blood after 24 h exposure to LPS, iron, LTA1 and LTA2			
Dunnett's multiple comparisons test	mean diff.	95.00% CI of diff.	adjusted *p*-value
*R*-value comparisons			
***R* naive versus *R* iron**	**2**.**219**	**1.197–3.242**	**<0**.**0001**
*R* naive versus *R* LPS	0.7023	−0.2922 to 1.697	0.24
*R* naive versus *R* LTA1	0.7756	−0.4489 to 2	0.33
*R* naive versus *R* LTA2	0.989	−0.2356 to 2.213	0.15
*K*-value comparisons			
*K* naive versus *K* iron	0.438	−0.1278 to 1.004	0.18
*K* naive versus *K* LPS	−0.1983	−0.7485 to 0.3519	0.78
*K* naive versus *K* LTA1	0.6283	−0.04913 to 1.306	0.08
*K* naive versus *K* LTA2	0.5217	−0.1558 to 1.199	0.18
**Angle comparisons**			
angle naive versus angle iron	−4.209	−9.765 to 1.347	0.12
angle naive versus angle LPS	2.563	−2.84 to 7.966	0.59
** angle naive versus angle LTA1**	**−6**.**737**	**−13.39 to −0.08449**	**0**.**05**
angle naive versus angle LTA2	−3.991	−10.64 to 2.662	0.4
**MA comparisons**			
MA naive versus MA iron	1.755	−2.594 to 6.105	0.71
MA naive versus MA LPS	1.345	−2.885 to 5.574	0.85
MA naive versus MA LTA1	−1.465	−6.674 to 3.743	0.89
MA naive versus MA LTA2	−0.4188	−5.627 to 4.789	0.99
**MRTG comparisons**			
MRTG naive versus MRTG iron	−0.3599	−1.428 to 0.708	0.82
MRTG naive versus MRTG LPS	0.2434	−0.7951 to 1.282	0.94
** MRTG naive versus MRTG LTA1**	**−1**.**334**	**−2.613 to −0.05524**	**0**.**04**
MRTG naive versus MRTG LTA2	−1.004	−2.283 to 0.2748	0.17
**TMRTG comparisons**			
** TMRTG naive versus TMRTG iron**	**3**.**094**	**1.52–4.667**	**<0**.**0001**
TMRTG naive versus TMRTG LPS	0.5904	−0.9399 to 2.121	0.74
TMRTG naive versus TMRTG LTA1	1.718	−0.1666 to 3.602	0.08
TMRTG naive versus TMRTG LTA2	1.608	−0.2766 to 3.492	0.12
**TTG comparisons**			
TTG naive versus TTG iron	71.23	−62.57 to 205	0.49
TTG naive versus TTG LPS	68.73	−61.4 to 198.9	0.49
TTG naive versus TTG LTA1	−55.51	−215.7 to 104.7	0.80
TTG naive versus TTG LTA2	−8.66	−168.9 to 151.6	0.99

Iron results show significant *p*-values for *R*, *K*, angle and TMRTG values, suggesting that the clot forms faster, the clot reaches its (20 mm) set point more slowly, there is more cross-linking of fibrin fibres and there is a decreased time from clot initiation to maximum clot formation. Considering that iron affects more TEG parameters than does LPS, free iron therefore has a more profound effect on clot formation than does LPS at the concentrations used, causing WB to be more hypercoagulable [8]. When LTA2 was used, only the MA and the TTG parameters of LTA2 were significantly different from the naive results.

Table 3 shows TEG analyses performed with one-way ANOVA and Dunnett's multiple comparisons analyses. Although this test is recommended for multiple samples, it compares the mean of each column with the mean of the control column. As discussed in Material and methods, we have unequal rows, and this had a slight impact on the *p*-values (table 3). However, the same hypercoagulable clot structure is confirmed.

Overall, the TEG results provide further evidence that the type of amyloid formed differs between LPS, iron, LTA1 and LTA2 when they are used as inducers.

## 4. Discussion

In previous work [11,12], as part of a systems biology approach to understanding the dormant bacterial basis and aetiology of coagulopathies accompanying various inflammatory diseases (e.g. [2,4–6,9,95,96]), we demonstrated that exceptionally low (and highly substoichiometric) concentrations of LPS could induce the formation of an amyloid form of fibrin. A chief piece of evidence was the extensive fluorescence staining when ThT was added. This was an entirely novel and unexpected finding, although Strickland and co-workers have shown that fibrin(ogen) can interact with known β-amyloid-forming peptides and proteins (e.g. [97–102]).

LPS is a component of Gram-negative bacteria, so an obvious question arose as to whether or not the equivalent molecules in Gram-positive organisms (especially LTA) would have similar effects. In addition, we wished to take the opportunity to assess the utility of various novel conjugated oligothiophene amyloid stains, now commercially available as the ‘Amytracker’™ series, to illuminate amyloids.

In the present paper, we show that LPS, iron and the two LTAs cause amyloid formation of plasma proteins, and in particular of fibrin(ogen) as blood is clotted. We confirmed amyloidogenesis by using small ligands identifying amyloid protein deposits [72]. Specifically, we used Amytracker™ 480 (related to HS163) and 680 (related to HS169) which are fluorescent amyloid ligands, also termed LCOs [72,78]. These two fluorescent markers bind rapidly and with high sensitivity to detect protein amyloid formation in fibrin(ogen). Previously, we showed that ThT binds to areas of amyloidogenic fibrin(ogen) that are created by LPS exposure during clotting. Here, we confirm that observation using LPS, and show further that iron and the two LTAs all cause changes in fibrin(ogen) conformation to an amyloid(ogenic) nature, with the role of iron being recognized in assisting the regrowth of dormant bacteria that can then shed the inflammagenic cell wall materials [11].

It is known that the Amytracker™ dyes are spectrally richer, can discriminate different forms of amyloid, and that their staining properties clearly differ from those of ThT [68,70,79–81]. Comparison (e.g. figure 2) of the staining with the three dyes (ThT and Amytracker™ 480 and 680) in the presence of the four candidate amyloidogenic molecules showed that this is also true for the amyloid form(s) of fibrin induced by the different agents. These clearly different staining patterns occur for each dye, with those of the two Amytracker™ dyes showing greater staining and being more similar (but not identical) to each other. Because the stoichiometry is of the order of 10^−8^ LPS/LTA : fibrinogen, we have been unable to determine the binding sites, though clearly the fact that they differ is the underlying cause of the different morphologies observed. As a referee pointed out, it is possible to view the binding of LPS/LTA by fibrinogen as a host-protective mechanism; however, the balance between the inflammatory potential of LPS/LTA and the amyloid forms of fibrin is, as yet, unknown.

This becomes even clearer when we observe the staining in the *z*-stack projections (figure 3; electronic supplementary material, video information of the clots shown in figure 3), with the Amytracker™ 480 seeming to favour the larger fibres characteristic of LTA2. These differences were also observed in the TEG traces using the paired *t*-test: while iron changed four of the TEG variables significantly (*R*, *K*, angle and TMRTG), LPS showed a significantly increased *R*-value, LTA1 was without effect, while LTA2 affected only the MA and TTG (and that marginally).

We conclude that both the LTA species' effects on the amyloid footprints, visible with confocal microscopy, are even more potent and effective than is LPS, in binding to fibrinogen and in affecting the manner in which it self-organizes during blood clotting. Such findings have profound significance for our understanding of the aetiology of anomalous blood clots, and may have value in diagnosis, prognosis and treatment of chronic, inflammatory diseases.

## Data accessibility

Raw data are stored on figshare.com: https://doi.org/10.6084/m9.figshare.5811138.
